# Whole transcriptome analysis of canine pheochromocytoma and paraganglioma

**DOI:** 10.3389/fvets.2023.1155804

**Published:** 2023-08-24

**Authors:** Marit F. van den Berg, Hans S. Kooistra, Guy C. M. Grinwis, Stefano Nicoli, Stefania Golinelli, Lisa Stammeleer, Monique E. van Wolferen, Elpetra P. M. Timmermans-Sprang, Maurice M. J. M. Zandvliet, Frank G. van Steenbeek, Sara Galac

**Affiliations:** ^1^Department of Clinical Sciences, Faculty of Veterinary Medicine, Utrecht University, Utrecht, Netherlands; ^2^Department of Biomolecular Health Sciences, Faculty of Veterinary Medicine, Utrecht University, Utrecht, Netherlands; ^3^Anicura Veterinary Clinic Roma Sud, Rome, Italy; ^4^Department of Veterinary Medical Science, Faculty of Veterinary Medicine, University of Bologna, Bologna, Italy; ^5^Small Animal Department, Faculty of Veterinary Medicine, Ghent University, Merelbeke, Belgium; ^6^Department of Cardiology, Division of Heart and Lungs, University Medical Center Utrecht, Utrecht, Netherlands

**Keywords:** pheochromocytoma, paraganglioma, adrenal, dog, transcriptome, RNA sequencing, therapeutic target, prognostic marker

## Abstract

Pheochromocytomas and paragangliomas (PPGLs) are neuroendocrine tumors arising from the chromaffin cells in the adrenal medulla and extra-adrenal paraganglia, respectively. Local invasion, concurrent disorders, and metastases prevent surgical removal, which is the most effective treatment to date. Given the current lack of effective medical treatment, there is a need for novel therapeutic strategies. To identify druggable pathways driving PPGL development, we performed RNA sequencing on PPGLs (*n* = 19) and normal adrenal medullas (NAMs; *n* = 10) of dogs. Principal component analysis (PCA) revealed that PPGLs clearly clustered apart from NAMs. In total, 4,218 genes were differentially expressed between PPGLs and NAMs. Of these, 232 had a log_2_ fold change of >3 or < −3, of which 149 were upregulated in PPGLs, and 83 were downregulated. Compared with NAMs, PPGLs had increased expression of genes related to the cell cycle, tumor development, progression and metastasis, hypoxia and angiogenesis, and the Wnt signaling pathway, and decreased expression of genes related to adrenal steroidogenesis. Our data revealed several overexpressed genes that could provide targets for novel therapeutics, such as Ret Proto-Oncogene (*RET*), Dopamine Receptor D2 (*DRD2*), and Secreted Frizzled Related Protein 2 (*SFRP2*). Based on the PCA, PPGLs were classified into 2 groups, of which group 1 had significantly higher Ki67 scores (*p* = 0.035) and shorter survival times (*p* = 0.04) than group 2. Increased expression of 1 of the differentially expressed genes between group 1 and 2, pleiotrophin (*PTN*), appeared to correlate with a more aggressive tumor phenotype. This study has shed light on the transcriptomic profile of canine PPGL, yielding new insights into the pathogenesis of these tumors in dogs, and revealed potential novel targets for therapy. In addition, we identified 2 transcriptionally distinct groups of PPGLs that had significantly different survival times.

## 1. Introduction

Pheochromocytomas and paragangliomas (PPGLs) are neuroendocrine tumors arising from the chromaffin cells in the adrenal medulla (pheochromocytomas, PCCs) or extra-adrenal paraganglia (paragangliomas, PGLs) (1, 2). When biochemically functional, they secrete excessive amounts of catecholamines, causing a variety of nonspecific, potentially fatal, clinical signs, such as tachyarrhythmias, hypertension, abdominal pain, and tachypnea. Clinical signs also can be related to the space-occupying or invasive nature of the mass (1, 3, 4).

Vascular invasion is observed in up to 82% of dogs with PPGL, while distant metastasis is reported in up to 24% of cases (4, 5). PCC and PGL are estimated to account for approximately 0.01–0.1 and 0.2%, respectively, of all canine tumors (1). This number is likely underestimated because it is primarily based on autopsy findings rather than the recently available biochemical testing. Currently, surgical resection is the treatment of choice for PPGLs in dogs and humans (1, 2). However, in some patients, surgical removal is prevented by local invasion, concurrent disorders, or distant metastases. In these patients, medical treatment is warranted.

The use of toceranib phosphate, a tyrosine kinase inhibitor (TKI), has been described in a small, retrospective series of 5 dogs with PCCs and 2 retrospective series of 27 and 28 dogs with PGLs, showing clinical benefit to some extent in the majority of dogs (6–8). However, most of these dogs only experienced stable disease as the best response, emphasizing the need for novel medical treatment options for canine PPGL.

The underlying mechanisms driving PPGL formation in humans are diverse, affecting a broad range of biological pathways, which is reflected in The Cancer Genome Atlas (TCGA) classification of PPGLs into 4 transcriptionally defined groups: the pseudohypoxia cluster, kinase signaling cluster, Wnt-altered cluster, and cortical admixture cluster (9). New therapeutic approaches in humans with PPGL are based on targeting these altered signaling pathways, as demonstrated by the use of hypoxia-inducible factor (HIF) inhibitors for tumors from the pseudohypoxia cluster (10–12) and the use of TKIs for metastatic tumors from the pseudohypoxia and kinase signaling cluster (2, 13, 14). In contrast, canine PPGL has not been well characterized on the molecular level, thus hampering the development of molecular-targeted therapies for these tumors in dogs.

Given the current lack of global gene expression data in canine PPGL and the lack of effective medical treatment for these tumors in dogs, we performed RNA sequencing on PPGLs and normal adrenal medullas (NAMs) of dogs, with 2 aims: (1) to unravel their transcriptomic signature, in this way yielding new insight into the pathogenesis of these tumors, and (2) to identify potential novel therapeutic targets.

## 2. Materials and methods

### 2.1. Patient population and sample collection

Tumor tissue was obtained from 21 client-owned dogs with PPGL (20 PCCs, 1 PGL) following surgical removal or immediately after euthanasia. Based on the principal component analysis (PCA) plot, 2 PPGLs were identified as outliers, which were excluded from further analyses, leaving a total of 19 PPGLs (18 PCCs, 1 PGL) in our final analysis cohort. Samples were collected at Utrecht University’s Department of Clinical Sciences, Faculty of Veterinary Medicine, for 8 dogs. In addition, samples were collected from contributing veterinary institutions, including University of Bologna (*n* = 2), Ghent University (*n* = 2), University of Ljubljana (*n* = 1), and veterinary referral centers in Italy (*n* = 2), Slovenia (*n* = 1), and the Netherlands (*n* = 3). Tumor tissues were cut longitudinally, and a representative piece of the tumor was either snap-frozen in liquid nitrogen or first fixed in RNA*later* stabilization solution (Invitrogen™, ThermoFisher Scientific, Breda, The Netherlands) and then stored at −70°C until RNA extraction. The remainder of the tissue was formalin-fixed and paraffin-embedded for histopathology and immunohistochemistry (IHC). In all dogs, the diagnosis of PPGL was confirmed by histopathology and IHC using the adrenomedullary markers chromogranin A (CHGA) and synaptophysin (SYP) (1, 15). Normal adrenal tissues were obtained from 10 healthy dogs. Adrenal glands were cut longitudinally, and the medullas were collected and snap-frozen.

Clinicopathological features, such as the presence of excessive catecholamine production, tumor location, tumor size, vascular invasion or the presence of a tumor thrombus, the presence of metastasis, survival time, histopathology results, and IHC results for CHGA, SYP, and Ki67, were recorded for all patients.

### 2.2. Ethical approval statement

Ethical approval was not required because the tissue samples originated from client-owned dogs with naturally occurring pheochromocytoma and paraganglioma, collected after surgical resection for curative purposes, or collected after euthanasia. Permission to use tissues for research purposes was obtained from all dog owners. Normal adrenal tissues were obtained from healthy dogs used in animal research, that were euthanized for reasons unrelated to the present study, which was approved by the Ethical Committee of Utrecht University.

### 2.3. Histopathology and immunohistochemistry

Histopathological examination by a veterinary pathologist (G.G.) and IHC for CHGA and SYP were performed for all canine samples. Slides were deparaffinized and rehydrated according to general histological techniques. For SYP IHC, antigen retrieval was performed with citrate buffer (pH 6) at 97°C for 20 min using PT link (Dako, Agilent Technologies). For CHGA IHC, no antigen retrieval was performed. Slides were incubated with Peroxidase Blocking Solution (Dako, Agilent Technologies) for 5 min to block endogenous peroxidase. Subsequently, slides were blocked with 10% normal goat serum in Tris buffered saline (TBS) for 15 min. Slides were incubated with primary antibodies for 60 min. The mouse monoclonal primary anti-CHGA antibody (ab199014, Abcam, Amsterdam, The Netherlands) was used in a 1:200 concentration, while the mouse monoclonal primary anti-SYP antibody (M731529-2, Agilent Technologies) was used in a 1:100 concentration. Incubation with a secondary antibody (HRP-labelled goat-anti-mouse, Brightvision, ImmunoLogic, WellMed, Duiven, The Netherlands) was performed at room temperature for 30 min. The slides were incubated with chromogen AEC (ab64252, Abcam) for 20 min, and counterstained with hematoxylin using Lab Vision Autostainer 360 (ThermoFisher Scientific). The slides were then rinsed in tap water and mounted with Aquatex (VWR International, Amsterdam, The Netherlands). During intermediate steps, the slides were washed in TBS with Tween-20 (Merck, Darmstadt, Germany) and bovine serum albumin (BSA). Normal canine pancreas with islets of Langerhans or normal canine adrenal medulla were used as positive controls for both CHGA and SYP, although often the histological samples contained pre-existing normal adrenal tissue that could serve as internal positive control (see Supplementary Presentation). Omission of the primary antibody from the immunohistochemical protocol was used as negative control. Cytoplasmatic staining of neoplastic cells for CHGA and SYP was evaluated by 2 observers (G.G. and M.v.d.B.).

Because the Ki67 proliferation index is used in humans with PPGL to assess malignant potential (16), Ki67 IHC is performed as standard of care for all canine PPGLs at our pathology department. Immunohistochemistry for Ki67 was performed as previously described (17), using a mouse monoclonal primary anti-Ki67 antibody (Agilent Cat# M7240, RRID: AB_2142367). Normal canine colon tissue slides were used as positive control, and the primary antibody was omitted for the negative control. The Ki67 score was assessed by 2 observers (M.v.d.B. and M.v.W.) in hot spot areas, in which the percentage of neoplastic cells with nuclear positivity was counted in a minimum of 1,000 cells. ImageJ software was used for this purpose (18). Care was taken to only include PPGL cells, and not areas of necrosis or hemorrhage. The Ki67 score was calculated as the percentage of Ki67 positive nuclei relative to the total number of counted nuclei for each PPGL. The inter-observer agreement was calculated and the average of the 2 Ki67 scores was used for further analyses.

### 2.4. RNA extraction, library preparation, and sequencing

RNA was isolated from PPGLs and NAMs using the RNeasy Mini Kit (Qiagen, Venlo, The Netherlands) following the manufacturer’s instructions. RNA samples were quantified using the Qubit™ RNA high sensitivity Assay Kit (ThermoFisher Scientific, Breda, The Netherlands). The quality of RNA samples was measured with RNA Nano Chips (Agilent Technologies, Middelburg, The Netherlands) on an Agilent Bioanalyzer 2,100 (Agilent Technologies). Samples with an RNA integrity number value <7.5 were excluded from further analysis.

A minimum of 200 ng of RNA from each sample was submitted for library preparation and sequencing at Utrecht Sequencing Facility. Sequencing libraries were made using the TruSeq Stranded mRNA Library Prep Kit (Illumina). Libraries were sequenced using the Nextseq2000 platform (Illumina), producing single-end reads of 50 bp. Reads were aligned to the canine reference genome CanFam3.1 using STAR version 2.4.2a.^1^ The raw and analyzed files have been uploaded to Gene Expression Omnibus under accession number GSE223526.

### 2.5. RNA sequencing data analysis

The dataset was normalized for composition bias using trimmed mean of M-values (TMM) and fold changes (FCs) were calculated in edgeR (19). Pairwise comparisons for differences in gene expression included NAM against PPGL and, among PPGL, group 1 against group 2, which were defined based on their PCA profile. The Mann–Whitney U-test was used to calculate *p*-values, which were corrected for multiple comparisons using the false discovery rate (FDR) Benjamini-Hochberg method. For the comparison between NAM and PPGL, genes were considered differentially expressed at an FDR-adjusted *p* < 0.05 and with a log_2_ FC of >3 or < −3. For the comparison between PPGL groups 1 and 2, genes were considered differentially expressed with the same cutoffs for the log_2_ FC, but at an FDR-adjusted *p* < 0.1. Volcano plots were generated using EnhancedVolcano (R package version 1.12.0) (20). A heatmap of differentially expressed genes (DEGs) was created using gplots.

#### 2.5.1. Pathway enrichment analysis

Pathway enrichment analysis was performed using Gene Set Enrichment Analysis 4.2.3 (21, 22). All unannotated genes were filtered, and the gene list of annotated genes was ranked by multiplying the sign of the log_2_ FC and − log10 of the FDR-adjusted *p* value for each gene, following best practices described in the literature (23). The database of pathway gene sets used for pathway enrichment analysis was downloaded from http://baderlab.org/GeneSets (Human_GOBP_AllPathways_no_GO_iea_August_2022_symbol.gmt) and contained pathways from 8 data sources: GO, Reactome, Panther, NetPath, NCI, MSigDB curated gene sets (C2 collection), MSigDB Hallmark (H collection), and HumanCyc (23). To ensure a robust analysis, pathways with an FDR-adjusted *p* ≤ 0.05 were considered significant.

### 2.6. Statistical analyses

Further data analysis was performed using the statistical software program IBM SPSS Statistics for Windows, version 27 (IBM Corp., Armonk, NY, United States). Data were assessed for normality of distribution using the Shapiro–Wilk test. For the comparison of clinicopathological features between PPGL group 1 and 2, differences for continuous and normally distributed data (Ki67 proliferation index, tumor diameter) were assessed using the independent samples t-test, whereas Fisher’s exact test was used for categorical data (tumor location, the presence of excessive catecholamine secretion, vascular invasion, the presence of metastasis). To compare pleiotrophin expression among multiple groups, the non-parametric Kruskal–Wallis test was used. In case of significance, differences between individual groups were assessed by *post hoc* analysis, and *p* values were adjusted by the Bonferroni correction method to adjust for multiple comparisons.

The inter-observer agreement score for Ki67 was quantified using the intra-class correlation coefficient for continuous variables. The strength of agreement was interpreted as follows: <0.40, poor; 0.40–0.59, moderate; 0.60–0.79, good; 0.80–1.00, excellent (17).

For the survival analyses, dogs were considered to have had an event when they had died or were euthanized due to PPGL attributable disease signs including metastases. Dogs that had died from a cause that appeared unrelated to PPGL, were lost to follow-up, or were still alive at the end of the study were censored at the timepoint they were last known to be alive. Survival times were recorded as the time between surgical resection of PPGL or initial diagnosis in patients that were not surgically managed, and censoring or death. Survival times were calculated using the Kaplan–Meier product-limit method. Significance between subpopulations was tested using the log-rank test for bivariate (categorical) variables. Values were considered significant at *p* < 0.05.

## 3. Results

### 3.1. Animals

The suspicion of PPGL was based on clinical signs, the presence of an adrenal (*n* = 18) or abdominal mass (*n* = 1) visualized by abdominal ultrasonography or computed tomography, increased plasma or urinary metanephrine concentrations (*n* = 10), and cytology following fine needle aspirations of the adrenal mass (*n* = 1). In 8 dogs, the adrenal mass was an incidental finding. The clinicopathological data of all included dogs can be found in Supplementary Table S1. The inter-observer agreement score for Ki67 was excellent (0.98; *p* < 0.001).

### 3.2. Differentially expressed genes

**Figure 1:**
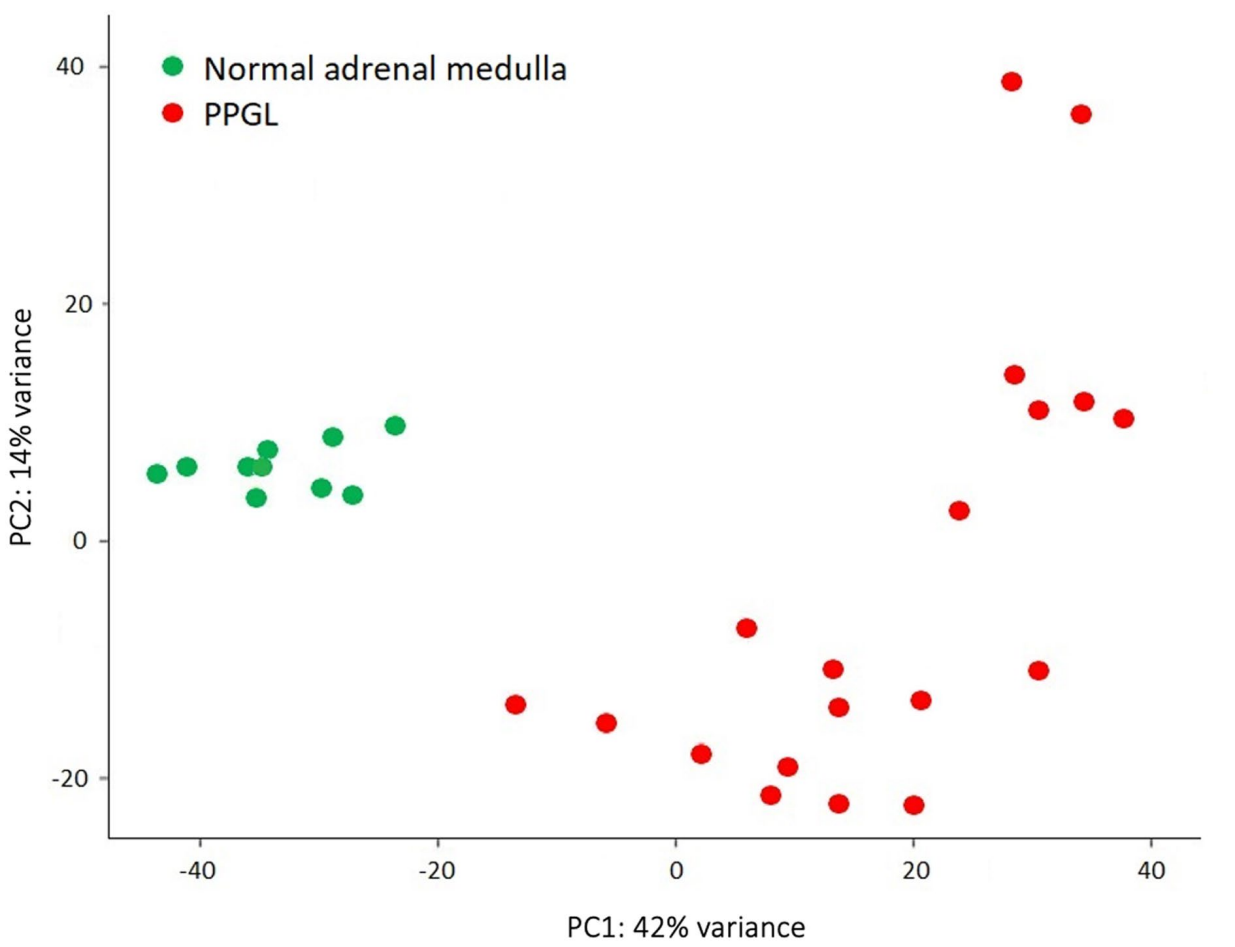
Principal component analysis shows that PPGLs clearly cluster apart from NAMs.

**Figure 2:**
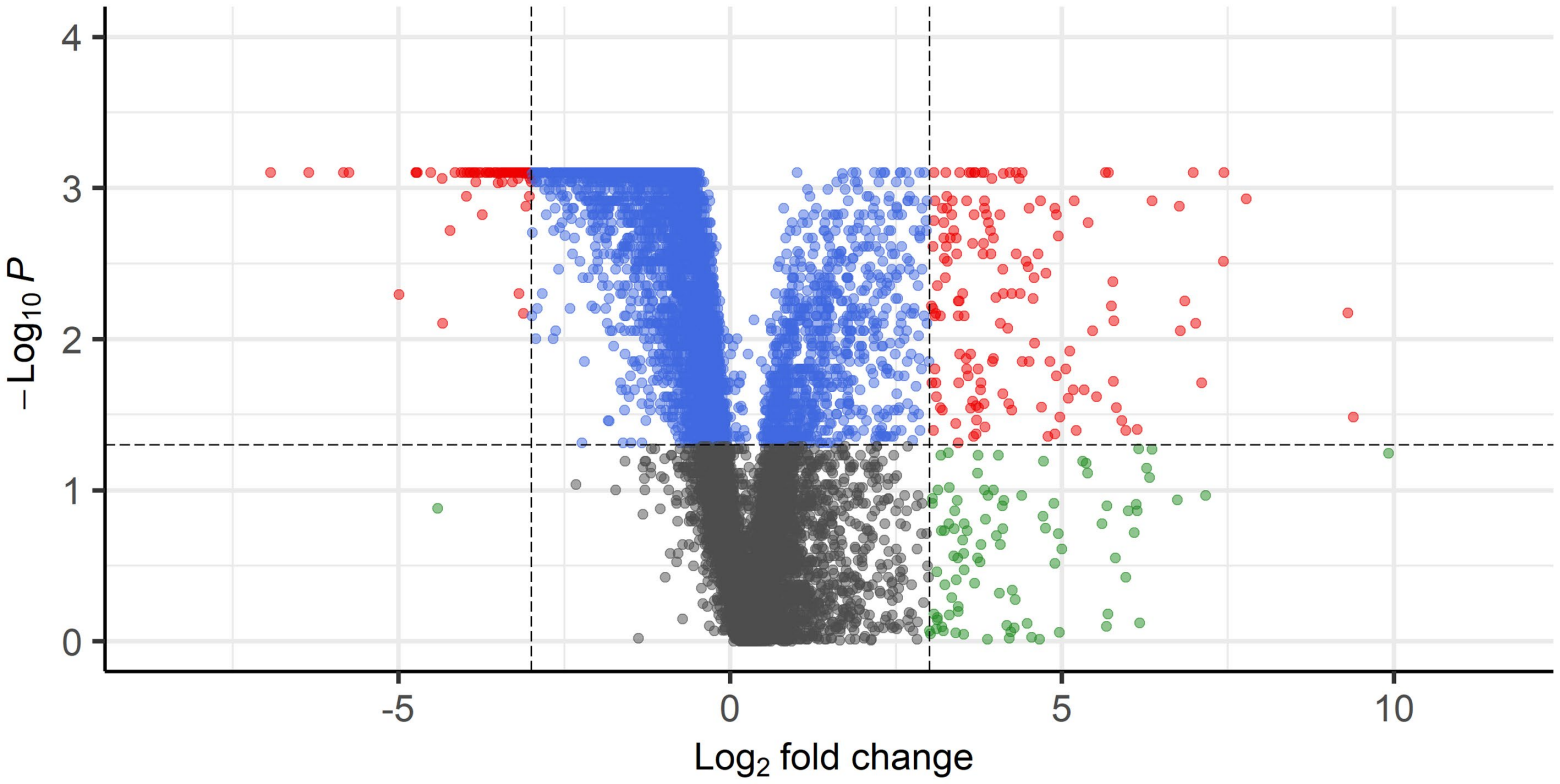
Volcano plot showing DEGs with FDR-corrected *p* < 0.05 and log_2_ FC of >3 (149 genes) or < −3 (83 genes) in red. Blue points represent genes with FDR-corrected *p* <0.05, but log_2_ FC of <3 and > −3, whereas gray points represent genes with FDR-corrected *p* > 0.05 and log_2_ FC of <3 and > −3. Genes with FDR-corrected *p* > 0.05 and log_2_ FC of >3 or < −3 are shown in green.

**Figure 3:**
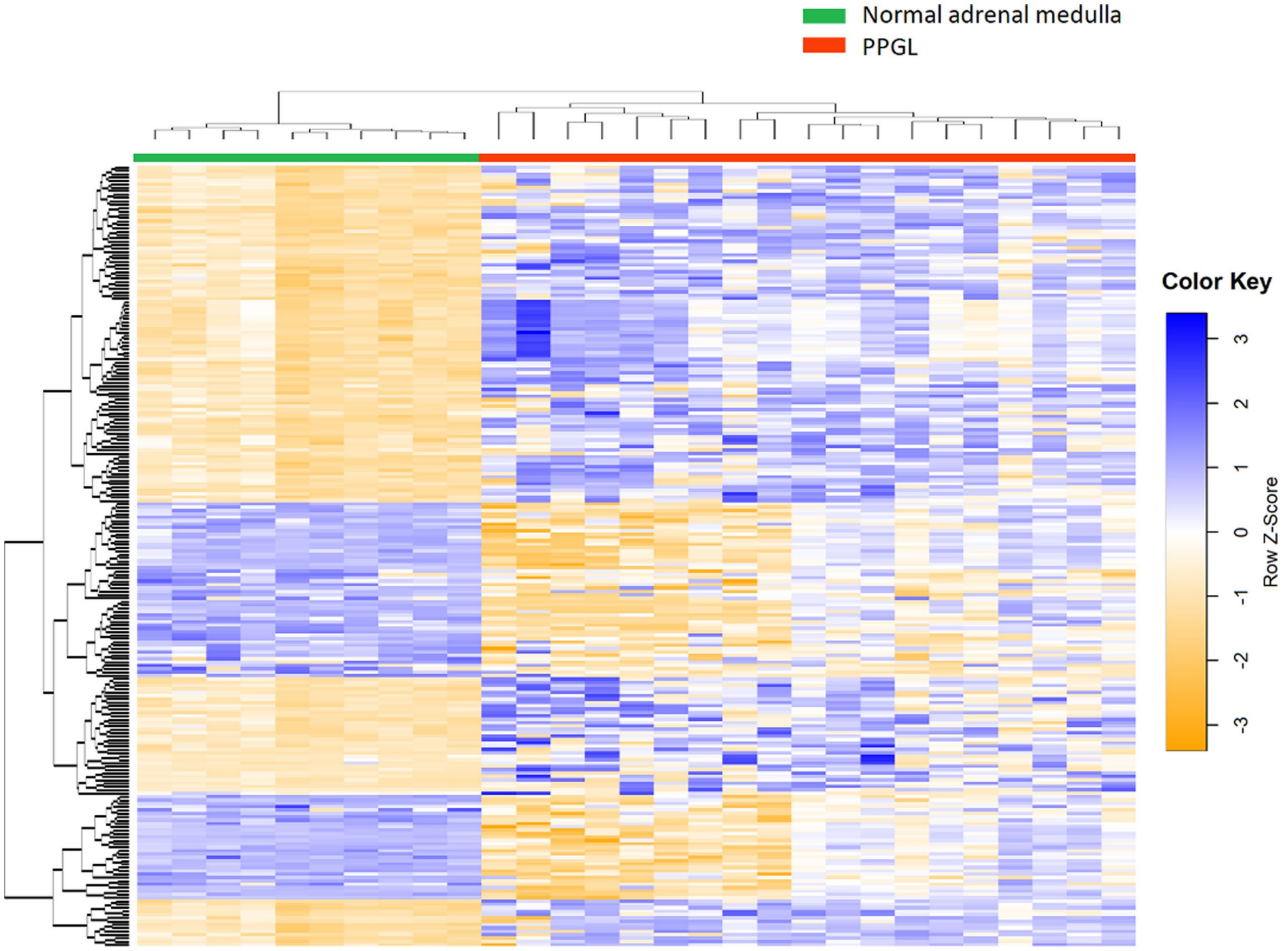
Heatmap of DEGs in PPGLs versus NAM. A total of 232 genes, with a log_2_ FC of >3 or < −3, are represented. Genes indicated in blue are upregulated, while genes in orange are downregulated.

Gene expression profiles were compared between PPGLs (*n* = 19) and NAMs (*n* = 10). In the PCA plot, PPGLs clearly clustered apart from NAMs (Figure 1), illustrating the differences in transcriptional behavior between tumor and normal samples. In total, 4,218 genes were differentially expressed between PPGLs and NAMs with an FDR-adjusted *p* < 0.05. Of these, 232 had a log_2_ FC of >3 or < −3, of which 149 were upregulated in PPGLs, and 83 were downregulated (Figure 2; Supplementary Tables S2, S3). Hierarchal clustering of these DEGs revealed distinct clustering of PPGL and NAM tissues (Figure 3). Table 1 shows the top 10 up-and downregulated genes based on their log_2_ FC.

**Table 1:** Top 10 up-and downregulated genes in PPGL versus NAM.

Ensembl ID	Gene symbol	Log_2_ fold change	FDR *p* value	Gene name
ENSCAFG00000002331	GFRAL	9.4	0.03265	GDNF family receptor alpha like
ENSCAFG00000031026	FOXI3	9.3	0.006683	Forkhead box I3
ENSCAFG00000008725	ADRA1A	7.8	0.001180	Adrenoceptor alpha 1A
ENSCAFG00000006073	POSTN	7.4	0.0007865	Periostin
ENSCAFG00000029224	HBM	7.4	0.003058	Hemoglobin subunit mu
ENSCAFG00000008353	SFRP2	7.1	0.01938	Secreted frizzled related protein 2
ENSCAFG00000017261	MEGF11	7.0	0.0007865	Multiple EGF like domains 11
ENSCAFG00000014218	NCAN	6.8	0.008809	Neurocan
ENSCAFG00000011198	ST8SIA2	6.8	0.001316	ST8 alpha-N-acetyl-Neuraminide alpha-2,8-sialyltransferase 2
ENSCAFG00000018461	TMEM255A	6.4	0.001215	Transmembrane protein 255A
ENSCAFG00000023388	UCN	−4.2	0.001904	Urocortin
ENSCAFG00000008440	FGG	−4.3	0.007857	Fibrinogen gamma chain
ENSCAFG00000007774	CELA1	−4.3	0.0008634	Chymotrypsin like elastase 1
ENSCAFG00000018002	KLHL14	−4.5	0.0007865	Kelch like family member 14
ENSCAFG00000006437	OXT	−4.7	0.0007865	Oxytocin
ENSCAFG00000003887	APOB	−4.7	0.0007865	Apolipoprotein B
ENSCAFG00000008591	TDO2	−5.0	0.005056	Tryptophan 2,3-dioxygenase
ENSCAFG00000001623	SLC24A2	−5.7	0.0007865	Solute carrier family 24 member 2
ENSCAFG00000031950	CARTPT	−5.8	0.0007865	Cocaine- and amphetamine-regulated transcript prepropeptide
ENSCAFG00000019126	CREB3L3	−6.4	0.0007865	cAMP responsive element binding protein 3-like 3

When researching these 232 up-and downregulated DEGs, data revealed that upregulated genes are associated with biological processes involving pro-tumorigenic factors, the cell cycle, tumor progression and metastasis, hypoxia and angiogenesis, and the Wnt signaling pathway (Table 2). Also, several upregulated genes, that are also highly overexpressed in human PPGL (e.g., Ret Proto-Oncogene [*RET*] (9), Dopamine Receptor D2 [*DRD2*] (24)), were identified. Many of the downregulated genes are involved in adrenal steroidogenesis. In addition, several genes that could serve as tumor suppressors are downregulated in PPGLs (Table 2). Table 3 shows a selection of overexpressed genes in canine PPGL that could provide targets for novel therapeutics.

**Table 2:** Examples of up-and downregulated DEGs between PPGL and NAM that are involved in different biological processes.

Upregulated genes in PPGL compared to NAM	
**Pro-tumorigenic factors**	
KIF11	Kinesin family member 11
FAP	Fibroblast activation protein alpha
**Cell cycle**	
PCLAF	PCNA clamp associated factor
CCNB2	Cyclin B2
CDC6	Cell division cycle 6
TOP2A	DNA topoisomerase II alpha
BUB1	BUB1 mitotic checkpoint serine/threonine kinase
**Tumor progression and metastasis**	
POSTN	Periostin
FOXI3	Forkhead box I3
MYBL2	MYB proto-oncogene like 2
**Hypoxia and angiogenesis**	
CDCP1	CUB domain containing protein 1
ANGPTL7	Angiopoietin like 7
EDN2	Endothelin 2
ESM1	Endothelial cell specific molecule 1
**Wnt signaling pathway**	
WNT3	Wnt family member 3
SFRP2	Secreted frizzled related protein 2
NKD1	NKD inhibitor of WNT signaling pathway 1
Downregulated genes in PPGL compared to NAM	
**Adrenal steroidogenesis**	
SF-1	Steroidogenic factor 1
SOAT1	Sterol O-acyltransferase 1
MCR2	Melanocortin 2 receptor
CYP21A2	Cytochrome P450 family 21 subfamily A member 2
CYP11A1	Cytochrome P450 family 11 subfamily A member 1
STAR	Steroidogenic acute regulatory protein
**Potential tumor suppressors**	
ERBB4	Erb-B2 receptor tyrosine kinase 4
CCBE1	Collagen and calcium binding EGF domains 1
MMRN1	Multimerin 1
GJA1	Gap junction protein alpha 1

**Table 3:** Overexpressed DEGs (log_2_ FC >3, FDR-adjusted *p* < 0.05) in dogs with PPGL that could provide targets for novel therapeutics.

Gene symbol	Gene name	Therapeutic strategies	Fold increase	References
RET	Ret proto-oncogene	Tyrosine kinase inhibitors, selective RET inhibitors	15	(13, 14, 25)
GFRAL	GDNF family receptor alpha like	Anti-GFRAL antibodies	672	(25, 26)
DRD2	Dopamine receptor D2	DRD2 antagonists	36	(27)
EDN2	Endothelin 2	Endothelin receptor antagonists	36	(28)
POSTN	Periostin	Anti-POSTN antibodies, POSTN-binding DNA aptamers	174	(29–31)
CDCP1	CUB domain containing protein 1	Anti-CDCP1 antibodies (± conjugated to cytotoxic agents), inhibition of CDCP1 molecular interactions	30	(32)
FAP	Fibroblast activation protein alpha	Chemotherapeutic drugs conjugated with a FAP substrate	23	(33)
ESM1	Endothelial cell specific molecule 1	Small interfering RNAs, short hairpin RNAs, microRNA (miR-9-3p), anti-ESM-1 antibodies	19	(34)
SFPR2	Secreted frizzled related protein 2	Anti-SFRP2 antibodies	138	(35)
MAPK12	Mitogen-activated protein kinase 12	MAPK12 inhibitors, statins	14	(36, 37)
MYBL2	MYB proto-oncogene like 2	Inhibitors of downstream target genes of MYBL2	11	(38)
PTN	Pleiotrophin	Receptor (ALK) antagonist, ribozyme-targeting PTN, microRNAs	19	(39–41)
KIF11	Kinesin family member 11	Kinesin (Eg5) inhibitors	8	(42)
TOP2A	DNA topoisomerase II alpha	TOP2A inhibitors/TOP2A directed chemotherapy	10	(43)

### 3.3. Pathway enrichment analysis

Using an FDR-adjusted *p* ≤ 0.05 for enrichment, pathway enrichment analysis revealed 28 overexpressed and 9 underexpressed pathways in PPGLs (Table 4). When comparing PPGL to NAM samples, steroid metabolism and the tricarboxylic acid (TCA) cycle are among the enriched underexpressed pathways, while the most enriched overexpressed pathways are involved in the cell cycle.

**Table 4:** Over-and underexpressed pathways in dogs with PPGL.

Overexpressed pathways	FDR *p* value	NES
Sister chromatid segregation	0.001	2.04
Nuclear chromosome segregation	<0.001	2.01
Mitotic sister chromatid segregation	<0.001	1.99
Nuclear division	0.001	1.96
Chromosome segregation	0.001	1.93
Mitotic nuclear division	0.001	1.93
Mitotic spindle assembly	0.002	1.89
Organelle fission	0.003	1.89
Regulation of mitotic sister chromatid separation	0.005	1.87
Regulation of nuclear division	0.004	1.87
Mitotic spindle organization	0.004	1.86
Regulation of mitotic metaphase/anaphase transition	0.004	1.86
Regulation of cytokinesis	0.005	1.85
Regulation of mitotic nuclear division	0.005	1.85
Regulation of metaphase/anaphase transition of cell cycle	0.007	1.84
Regulation of chromosome separation	0.015	1.81
Plk1 signaling events	0.021	1.79
Negative regulation of sister chromatid segregation	0.024	1.78
Negative regulation of mitotic sister chromatid segregation	0.025	1.78
Mitotic spindle checkpoint signaling	0.026	1.78
Smooth muscle contraction	0.029	1.77
Negative regulation of chromosome segregation	0.034	1.76
Negative regulation of mitotic metaphase/anaphase transition	0.045	1.75
Negative regulation of chromosome separation	0.046	1.74
Kinesins	0.044	1.74
Negative regulation of metaphase/anaphase transition of cell cycle	0.043	1.74
Mitotic spindle assembly checkpoint signaling	0.043	1.74
Regulation of sister chromatid segregation	0.044	1.74
Underexpressed pathways	FDR *p* value	NES
Steroid metabolic process	0.001	−1.80
Organic acid catabolic process	0.017	−1.75
Transition metal ion transport	0.036	−1.72
Visual phototransduction	0.021	−1.71
Metabolism of vitamins and cofactors	0.024	−1.71
The citric acid (TCA) cycle and respiratory electron transport	0.027	−1.71
Carboxylic acid catabolic process	0.033	−1.71
Negative regulation of hemopoiesis	0.05	−1.68
Negative regulation of leukocyte differentiation	0.05	−1.65

The normalized enrichment score (NES) reflects the enrichment of the pathways at the top (overexpressed pathways) or at the bottom (underexpressed pathways) of the ranked gene list.

### 3.4. PPGL classification

**Figure 4:**
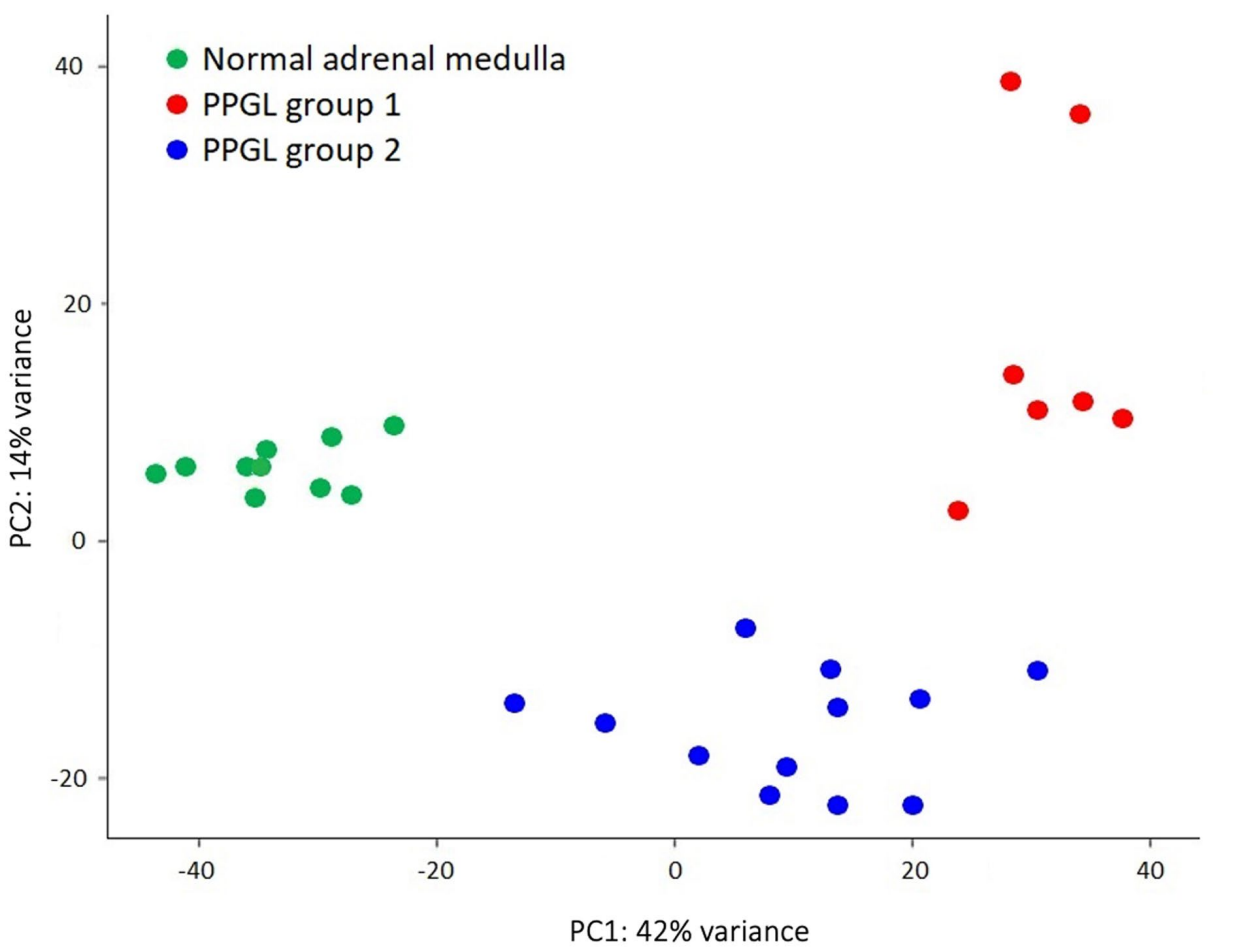
PPGL samples were classified based on their PCA profile as PPGL group 1 (*n* = 7) and PPGL group 2 (*n* = 12).

**Figure 5:**
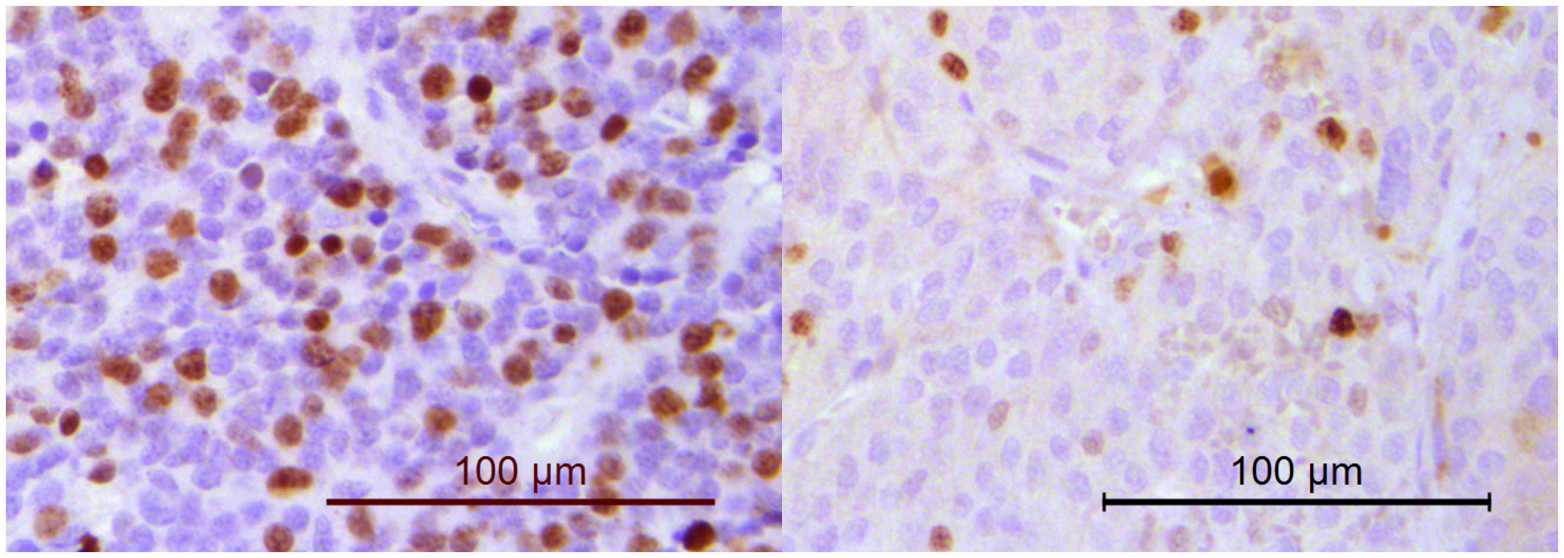
Immunohistochemistry for Ki67. The percentage of neoplastic cells with nuclear positivity was counted in a minimum of 1,000 cells. Dogs in PPGL group 1 (example on the left; Ki67 score of 33.4%) had significantly higher Ki67 scores than dogs in PPGL group 2 (example on the right; Ki67 score of 10.1%).

**Figure 6:**
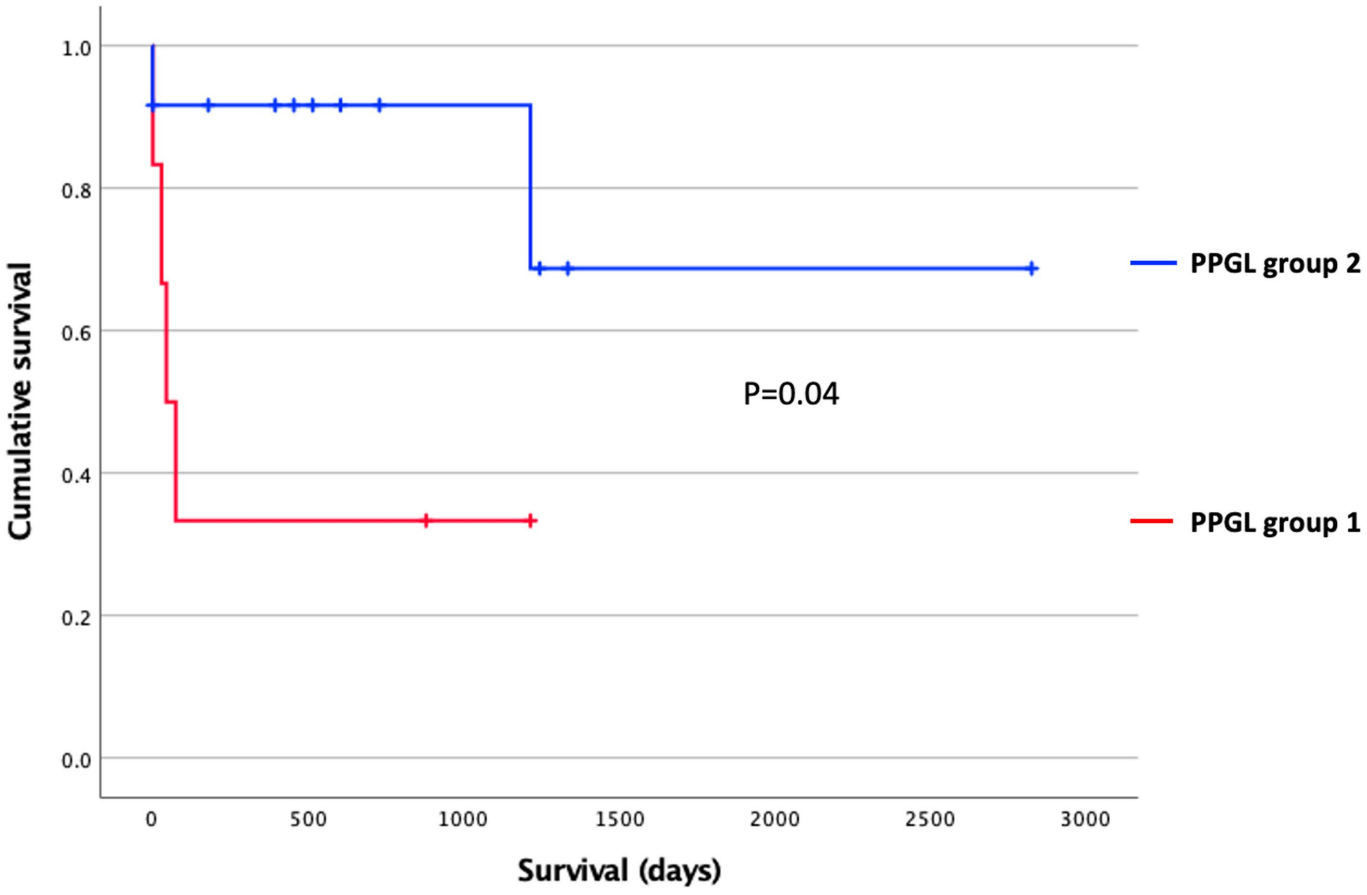
Survival stratified according to the PPGL classification using Kaplan–Meier analysis. Censored dogs are indicated as tick marks. The *p* value indicates the significance of the difference between PPGL group 1 and 2 as calculated with the log-rank test.

Another aim of this study was to evaluate whether subclustering was detectable among dogs with PPGL resulting in different gene expression profiles. We divided dogs with PPGL into 2 groups based on their PCA profile: PPGL group 1 (*n* = 7) and PPGL group 2 (*n* = 12) (Figure 4). When comparing these 2 groups based on their clinicopathological parameters, no significant differences between the 2 groups in tumor location (*p* = 0.78), tumor size (*p* = 0.16), the presence of excessive catecholamine secretion (*p* = 0.46), vascular invasion (*p* = 0.31), or the presence of metastasis (*p* = 0.34) were found. However, a significantly higher Ki67 score was found in group 1 (*p* = 0.035; Figure 5). In addition, dogs in group 1 had significantly (*p* = 0.04) shorter survival times (Figure 6), suggesting a more aggressive phenotype of tumors in group 1. For dogs in group 1, median overall survival time was 45 days (95% confidence interval [CI]: 0–99 days), while the median survival time for dogs in group 2 was not reached. The mean overall survival was 1,789 days (95% CI: 1,125–2,453 days), while mean overall survival was 430 days (95% CI: 0–875 days) and 2,221 days (95% CI: 1,489–2,953 days) for dogs in group 1 and group 2, respectively. Since 4 dogs in group 1 and 1 dog in group 2 did not receive treatment for their tumor, this could have affected the difference in survival times between the two groups. Survival analysis on those dogs that were surgically treated, showed a significantly (*p* = 0.019) shorter survival time for dogs in group 1 compared to group 2.

### 3.5. PPGL group 1 versus group 2

**Figure 7:**
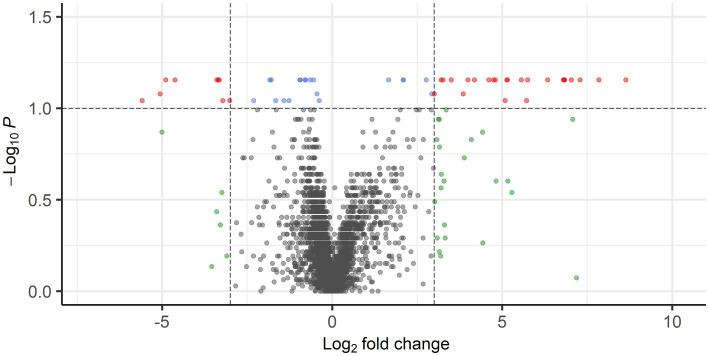
Volcano plot showing DEGs between PPGL group 1 and 2 with FDR-corrected *p* < 0.1 and log_2_ FC of >3 (24 genes) or <−3 (9 genes) in red.

When comparing PPGL group 1 with PPGL group 2, no genes were differentially expressed if an FDR-adjusted *p* < 0.05 was used. Because of the exploratory nature of this work, we increased the cut-off to an FDR-adjusted *p* < 0.1, which showed a total of 52 DEGs. Of these, 33 had a log_2_ FC of >3 (*n* = 9) or <−3 (*n* = 24) in PPLG group 1 compared to PPGL group 2 (Figure 7; Supplementary Table S4). Table 5 shows the upregulated and top 10 downregulated genes based on their log_2_ FC.

**Table 5:** Upregulated (*n* = 9) and top 10 downregulated genes in PPGL group 1 versus group 2.

Ensembl ID	Gene symbol	Log_2_ fold change	FDR *p* value	Gene name
ENSCAFG00000005036	DPP6	5.6	0.09083	Dipeptidyl peptidase like 6
ENSCAFG00000018449	ISL1	5.1	0.08352	ISL LIM homeobox 1
ENSCAFG00000003526	BRINP1	4.9	0.06994	BMP/retinoic acid inducible neural specific 1
ENSCAFG00000003369	PTN	4.6	0.06994	Pleiotrophin
ENSCAFG00000028604	VSTM2L	3.4	0.06994	V-Set and transmembrane domain-containing protein 2-like protein
ENSCAFG00000014616	COMP	3.3	0.06994	Cartilage oligomeric matrix protein
ENSCAFG00000009354	TOX2	3.3	0.06994	TOX high mobility group box family member 2
ENSCAFG00000009385	CHST1	3.2	0.09083	Carbohydrate sulfotransferase 1
ENSCAFG00000001178	COL22A1	3.0	0.09083	Collagen type XXII alpha 1 chain
ENSCAFG00000020362	HSD11B2	−5.7	0.09083	Hydroxysteroid 11-beta dehydrogenase 2
ENSCAFG00000031682	GSTA2	−5.7	0.06994	Glutathione S-transferase A2
ENSCAFG00000017950	LMAN1L	−6.3	0.06994	Lectin, mannose binding 1 like
ENSCAFG00000010039	HSD3B2	−6.8	0.06994	Hydroxy-delta-5-steroid dehydrogenase, 3 beta- and steroid delta-isomerase 2
ENSCAFG00000010279	CYP17A1	−6.8	0.06994	Cytochrome P450 family 17 subfamily A member 1
ENSCAFG00000000712	CYP21A2	−6.8	0.06994	Cytochrome P450 family 21 subfamily A member 2
ENSCAFG00000017888	CYP11A1	−7.0	0.06994	Cytochrome P450 family 11 subfamily A member 1
ENSCAFG00000001285	CYP11B2	−7.3	0.06994	Cytochrome P450 family 11 subfamily B member 2
ENSCAFG00000016269	NWD2	−7.8	0.06994	NACHT and WD repeat domain containing 2
ENSCAFG00000013466	LRRC15	−8.6	0.06994	Leucine rich repeat containing 15

When interrogating these up-and downregulated DEGs, data revealed that upregulated genes in group 1 were associated with cancer development and progression (e.g., ISL LIM Homeobox 1 [*ISL1*], Cartilage Oligomeric Matrix Protein [*COMP*], pleiotrophin [*PTN*]), which fits with the more aggressive phenotype of group 1. Many downregulated genes were involved in adrenal steroidogenesis (e.g., Hydroxysteroid 11-beta Dehydrogenase 2 [*HSD11B2*], Cytochrome P450 Family 11 Subfamily B Member 2 [*CYP11B2*] and Subfamily A Member 1 [*CYP11A1*], Cytochrome P450 Family 17 Subfamily A Member 1 [*CYP17A1*], Cytochrome P450 Family 21 Subfamily A Member 2 [*CYP21A2*], Steroidogenic Factor 1 [*SF-1*]). Pathway enrichment analysis revealed 4 underexpressed pathways (Table 6) in PPGL group 1 compared to group 2, but no overexpressed pathways when using an FDR-adjusted *p* ≤ 0.05 for enrichment.

**Table 6:** Underexpressed pathways in dogs from PPGL group 1 compared to group 2.

Underexpressed pathways	FDR *p* value	NES
Cellular ketone metabolic process	0.007	−1.69
Diseases of metabolism	0.008	−1.69
Steroid metabolic process	0.016	−1.69
Metabolism of steroids	0.011	−1.68

The NES reflects the enrichment of the pathways.

**Figure 8:**
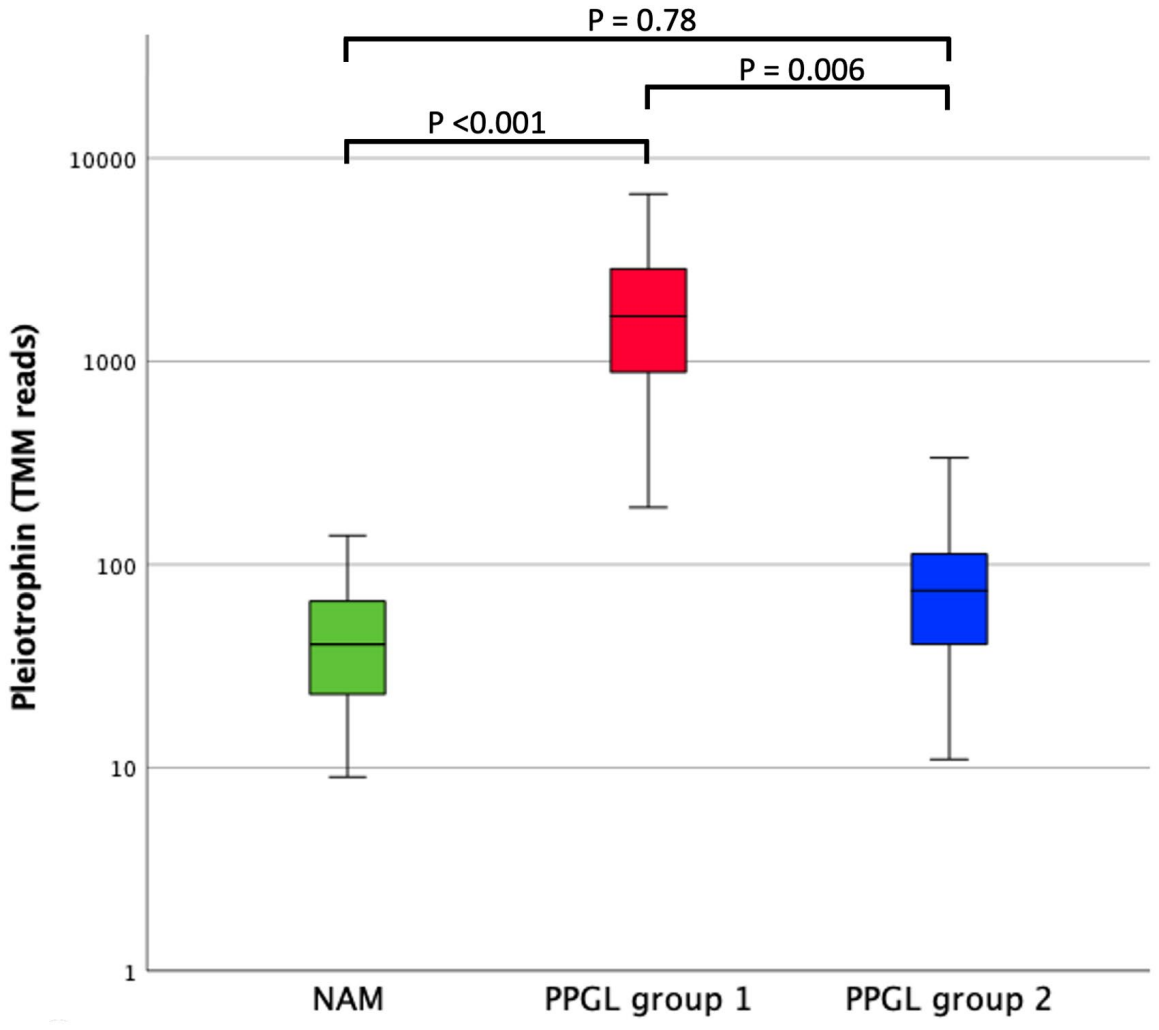
Comparison of pleiotrophin expression (TMM RNA sequencing reads) between NAM, PPGL group 1, and PPGL group 2. The box represents the inter quartile range (i.e., the second and third quartiles). The line within the box represents the median. The whiskers represent the range, extending to a maximum of 1.5-times the interquartile range.

In search for a therapeutic target that could specifically benefit patients with a poor prognosis (PPGL group 1), we compared DEGs between PPGLs and NAMs, and between PPGL group 1 and 2. When using an FDR-corrected *p* < 0.05 for the comparison between PPGL and NAMs, and an FDR-corrected *p* < 0.1 for the comparison between PPGL group 1 and 2, we found 1 gene that was upregulated with a log_2_ FC >3 and 8 genes that were downregulated with a log_2_ FC < -3 in both comparisons (Table 7). The expression of *PTN* was significantly higher in PPGL group 1 compared to NAM (*p* < 0.001), and in PPGL group 1 compared to group 2 (*p* = 0.006; Figure 8), which makes it a promising therapeutic target for patients with a poor prognosis.

**Table 7:** Genes that are up-or downregulated in PPGL compared to the NAM, as well as in PPGL group 1 compared to group 2.

Ensembl ID	Gene symbol	PPGL/NAM		PPGL 1/PPGL 2		Gene name
		Log_2_ FC	FDR *p* value	Log_2_ FC	FDR *p* value	
ENSCAFG00000003369	PTN	4.2	0.0296	4.6	0.0699	Pleiotrophin
ENSCAFG00000004615	APOC1	−3.1	0.000786	−4.0	0.0699	Apolipoprotein C1
ENSCAFG00000012374	FGFR2	−3.3	0.000786	−4.2	0.0699	Fibroblast growth factor receptor 2
ENSCAFG00000014189	ACSBG1	−3.1	0.000786	−5.1	0.0908	Acyl-CoA synthetase bubblegum family member 1
ENSCAFG00000023086	SF-1	−3.3	0.000786	−5.2	0.0699	Steroidogenic factor 1
ENSCAFG00000000865	ENPP2	−3.8	0.000786	−5.6	0.0699	Ectonucleotide pyrophosphatase/phosphodiesterase 2
ENSCAFG00000031682	GSTA2	−3.5	0.000786	−5.7	0.0699	Glutathione S-transferase A2
ENSCAFG00000000712	CYP21A2	−3.2	0.000786	−6.8	0.0699	Cytochrome P450 family 21 subfamily A member 2
ENSCAFG00000017888	CYP11A1	−3.4	0.000786	−7.0	0.0699	Cytochrome P450 family 11 subfamily A member 1

## 4. Discussion

This is the first study in which a transcriptome sequencing approach was applied to identify gene expression characteristics of canine PPGL. We have detected several genes and biological pathways that were dysregulated in PPGLs compared to NAMs, yielding new insights into the pathogenesis of these tumors in dogs and providing potential targets for therapeutic intervention. In addition, we identified 2 transcriptionally distinct groups of PPGLs that had significantly different survival times.

We detected 4,218 DEGs between NAM and PPGL, and hierarchal clustering of these DEGs revealed well-defined clustering of normal and tumor tissues, showing a distinct gene expression profile. Compared with healthy controls, dogs with PPGL had increased expression of genes related to the cell cycle, tumor development, progression and metastasis, hypoxia and angiogenesis, and the Wnt signaling pathway, and decreased expression of genes related to adrenal steroidogenesis.

Aberrant activation of the Wnt pathway is implicated in cancer initiation and progression (44). In humans with PPGL, the Wnt-altered cluster consists of adrenal PCCs with Mastermind Like Transcriptional Coactivator 3 (*MAML3*) fusion genes and Cold Shock Domain Containing E1 (*CSDE1*) somatic mutations, resulting in an activation of the Wnt and Hedgehog signaling pathways and overexpression of genes such as Wnt Family Member 4 (*WNT4*) and Dishevelled Segment Polarity Protein 3 (*DVL3*) (9). Although these specific genes are not differentially expressed between canine PPGL and NAM, we did find significantly increased expression of Wnt Family Member 3 (*WNT3*; 21-fold overexpressed), Secreted Frizzled Related Protein 2 (*SFRP2*; 138-fold overexpressed), and NKD Inhibitor of WNT Signaling Pathway 1 (*NKD1*; 22-fold overexpressed). Overexpression of *WNT3* activates the canonical Wnt pathway, and WNT3 treatment enhanced cell proliferation in rat PCC (PC12) cells (45). *SFRP2* can function both as a Wnt antagonist and agonist, whereas *NKD1* functions as an inhibitor of the Wnt signaling pathway (35). The role of *SFRP2* as enhancer of the Wnt signaling pathway, resulting in the upregulation of *WNT3*-regulated genes including its antagonist *NKD1*, has been previously described (46). Additionally, *SFRP2* has been shown to play a key role in tumor angiogenesis (35). Thus, the Wnt pathway, and specifically *SFRP2*, might show great promise as target for anti-cancer therapy in dogs with PPGL.

Besides genes related to the Wnt signaling pathway, our data revealed several other interesting genes that could provide targets for therapeutic intervention (also see Table 2). For example, the transcription factor MYB Proto-Oncogene Like 2 (*MYBL2*) was significantly overexpressed (11-fold) in PPGLs. *MYBL2* is an important physiological regulator of cell cycle progression and cell survival and promotes cancer initiation and/or progression when overexpressed (38). In humans with PPGL, increased *MYBL2* expression is significantly correlated with lower 5-years progressive-free interval (47). Although direct MYBL2 inhibitors are not yet available, inhibitors interfering with downstream target genes of MYBL2 may serve as effective novel therapeutics (38). We also noted increased (14-fold) expression of the gene Mitogen-Activated Protein Kinase 12 (*MAPK12*), which has been shown to play a role in cancer development, invasion, and metastasis (36). In humans with PPGL, the MAPK pathway is mainly associated with tumors from the kinase signaling cluster, but several MAPK-related genes, including *MAPK12*, are also upregulated in Succinate Dehydrogenase (SDH) B (*SDHB*)-derived PPGLs (37). It has been shown that treatment of mouse PCC cell lines with statins induced apoptosis by MAPK inhibition (37). Thus, treatment with specific MAPK12 inhibitors or statins might provide a promising treatment option for dogs with PPGL.

In humans with PPGL, several TKIs, including sunitinib, are currently under evaluation in phase II clinical trials^2^. Sunitinib targets multiple tyrosine kinase receptors, including RET, vascular endothelial growth factor receptors (VEGFR) 1 and 2, platelet-derived growth factor receptor (PDGFR)-β, and c-KIT (13, 14). Tumors from the pseudohypoxia cluster are characterized by activation of pseudohypoxic pathways with resultant activation of HIF target genes implicated in angiogenesis, including VEGF and PDGF (48). Thus, patients from the pseudohypoxia group may derive great benefit from sunitinib treatment (13). In addition, sunitinib may have additional activity in patients with increased RET expression (13). In our canine patients, we did not find increased expression of *VEGF*, *PDGF,* and their receptors at the mRNA level. In contrast, *RET* was significantly overexpressed (15-fold) in dogs with PPGL. Targets of the veterinary available TKI toceranib phosphate include VEGFR, PDGFR, C-KIT, and likely also RET, based on its structural and functional similarity to sunitinib (49). Although these findings should be validated by functional (i.e., protein expression) studies, this suggests that dogs with PPGL might benefit from a new generation of kinase inhibitors with improved selectivity for RET.

In this study, GDNF Family Receptor Alpha Like (*GFRAL*), which is the receptor for Growth Differentiation Factor 15 (*GDF15*), was the most upregulated gene in dogs with PPGL (672-fold increased). Of interest, signaling of glial-derived neurotrophic factor (GDNF)-family receptors occurs through the RET receptor tyrosine kinase (25, 50). GDNF-mediated activation of RET is recognized in several cancers and promotes tumor growth, invasion, and metastasis (25). Moreover, GDF15 is considered the main actor of cachexia in cancer signaling through its receptor GFRAL (26). Therefore, strategies targeting the GDF15-GFRAL-RET axis might be of interest to dogs with PPGL, both by their direct anti-tumor effect and by reducing cancer cachexia.

According to TCGA data, human PPGLs have the highest *DRD2* mRNA expression of all cancers. A phase II clinical trial of ONC201, a DRD2 antagonist, in human patients with metastatic neuroendocrine tumors including PPGL showed clinical benefit in the majority of patients with PPGL (5 partial responses, 7 stable diseases, 2 progressive diseases) (27). In our canine population, *DRD2* was significantly overexpressed (36-fold). Thus, the use of DRD2 antagonist might be beneficial to dogs with PPGL as well.

The decreased expression of adrenal steroidogenesis-related genes and gene pathways in PPGLs compared to NAMs can be explained by contamination of the NAM samples by adjacent cortical cells. PPGLs also showed decreased expression of the TCA cycle pathway. In humans, the pseudohypoxia cluster partially consists of tumors with mutations in TCA cycle-related genes, such as the SDH subunits (*SDHA/B/C/D*) and fumarate hydratase (2). In dogs with PPGL, mutations in *SDHB* and *SDHD* have been described (51, 52), suggestive of common pseudohypoxic pathways. The decreased expression of the TCA cycle pathway in PPGL dogs in our study may reflect this. Future studies on whole-exome or whole-genome sequencing will be needed to fully unravel the genetic background of canine PPGL.

The levels of expression of DEGs were not validated by quantitative real-time reverse transcription polymerase chain reaction (qRT-PCR) in this study, as the added value of this practice has been questioned recently. It has been shown that the vast majority of ‘non-concordant’ results between RNA sequencing and qRT-PCR analysis (defined as either both approaches leading to differential expression in opposite directions or one of the methods demonstrating differential expression while the other does not) is seen in genes with a log_2_ fold change of <2 (53), whereas a cutoff of a log_2_ fold change of >3 or < −3 was used in the present study for genes to be considered differentially expressed.

When comparing gene expression profiles among dogs with PPGL, DEGs only were identified at an FDR-adjusted *p* < 0.1, suggesting less transcriptomic variance between tumor tissues classified as PPGL group 1 versus group 2 than between PPGL and NAM. However, this also can be related to the small sample size and heterogeneous groups of patients. The less stringent FDR-adjusted *p* values should be taken into consideration regarding the robustness of the comparisons between PPGL group 1 and 2. Despite this, the classification of PPGL samples based on their position in the PCA plot did allow for the prognostic prediction of dogs with PPGL, showing significantly higher Ki67 scores and worse survival times of tumors in group 1. The survival analyses in this study, however, do have some limitations. In some patients, the cause of death or reason for euthanasia was presumed to be unrelated to PPGL based on the presence of unrelated clinical signs. To definitively exclude this, necropsy would have been required, which was not performed in the majority of dogs. Although a difference in treatment between dogs in group 1 and 2 was excluded as the reason for the shorter survival times of dogs in group 1, survival in patients undergoing surgical resection of PPGL might have been influenced by a combination of factors other than the tumor (such as expertise of the surgeon and anesthetist).

Compared to group 1, PPGL group 2 overexpressed many genes involved in adrenal steroidogenesis. In humans, the cortical admixture cluster is enriched for adrenal cortical markers such as Steroidogenic Acute Regulatory Protein (*STAR*), *CYP11B2,* and *CYP21A2*, which could represent adrenal cortical contamination as an artifact due to impure tumor sampling or could represent a unique distinct tumor biology (9, 54, 55). In our study, most dogs in PPGL group 1 presented with a tumor thrombus (Supplementary Table S1). Sampling of the tumor thrombus, instead of the adrenal mass itself, might have resulted in less contamination with cortical cells in these dogs, possibly explaining these differences between the 2 groups.

Currently, there is no reliable way to predict the metastatic potential of PPGLs in dogs. A previous study evaluated The Pheochromocytoma of the Adrenal Gland Scaled Score (PASS) and several IHC markers in dogs with PCCs, which were shown not to be associated with survival (56). In our study, increased expression of *PTN* appeared to correlate with a more aggressive tumor phenotype, which might suggest that *PTN* could have prognostic value in dogs with PPGL. The possibility that *PTN* might be an interesting prognostic marker needs to be evaluated in a larger cohort of patients with long-term follow-up, and, ideally, by functional studies. In humans, the expression of PTN is increased in several types of cancer, and overexpression is usually associated with a poor prognosis (39, 40, 57). According to the GEPIA database^3^, *PTN* is highly expressed in human PPGL. To determine whether *PTN* could also be a prognostic factor in human PPGL, it would be interesting to consult human RNA sequencing datasets with long-term follow-up. In a study of men with prostate cancer, serum-based PTN was found to be a biomarker for metastatic progression (58). Thus, PTN might provide a promising cancer liquid biopsy marker, which warrants further research to evaluate the utility of PTN as a clinical test in different types of cancer. In addition, PTN may be therapeutically targeted, for example by treatment with microRNAs, anti-PTN antibodies, ribozyme-mediated knockdown, or inhibition of its receptor anaplastic lymphoma kinase (ALK) (39, 59, 60).

In conclusion, this study has shed light on the transcriptomic profile of canine PPGL and revealed novel targets for therapy. Future *in vitro* and *in vivo* studies are warranted to determine whether mentioned drugs (such as DRD2 antagonists, MAPK12 inhibitors, RET inhibitors, or drugs targeting PTN) can inhibit tumor growth and improve survival and quality of life in dogs with PPGL.

## Data availability statement

The raw and analyzed datasets for this study can be found in online repository: NCBI GEO under accession number GSE223526.

## Ethics statement

Ethical approval was not required for the studies involving animals in accordance with the local legislation and institutional requirements because ethical approval was not required because the tissue samples originated from client-owned dogs with naturally occurring pheochromocytoma and paraganglioma, collected after surgical resection for curative purposes, or collected after euthanasia. Permission to use tissues for research purposes was obtained from all dog owners. Normal adrenal tissues were obtained from healthy dogs used in animal research, that were euthanized for reasons unrelated to the present study, which was approved by the Ethical Committee of Utrecht University. Written informed consent was obtained from the owners for the participation of their animals in this study.

## Author contributions

MB, FS, and SGa contributed to conception and design of the study. MB, HK, SN, SGo, LS, MW, ET-S, and SGa contributed to data acquisition. MB, GG, MW, FS, MZ, and SGa contributed to data analysis and interpretation. MB wrote the first draft of the manuscript. FS wrote sections of the manuscript. All authors contributed to the article and approved the submitted version.

## Funding

This study was supported by the foundations called ‘Stichting Abri voor Dieren’ and ‘Friends of VetMed’. The open access publication fee is funded by Utrecht University’s Faculty of Veterinary Medicine.

## Conflict of interest

The authors declare that the research was conducted in the absence of any commercial or financial relationships that could be construed as a potential conflict of interest.

## Publisher’s note

All claims expressed in this article are solely those of the authors and do not necessarily represent those of their affiliated organizations, or those of the publisher, the editors and the reviewers. Any product that may be evaluated in this article, or claim that may be made by its manufacturer, is not guaranteed or endorsed by the publisher.

## Figures and Tables

**Table:** 

BSA	Bovine serum albumin
CHGA	Chromogranin A
CI	Confidence interval
COMP	Cartilage oligomeric matrix protein
CSDE1	Cold shock domain containing E1
CYP11A1	Cytochrome P450 family 11 subfamily A member 1
CYP11B2	Cytochrome P450 family 11 subfamily B member 2
CYP17A1	Cytochrome P450 family 17 subfamily A member 1
CYP21A2	Cytochrome P450 family 21 subfamily A member 2
DEG	Differentially expressed gene
DRD2	Dopamine receptor D2
DVL3	Dishevelled segment polarity protein 3
FC	Fold change
FDR	False discovery rate
GDF15	Growth differentiation factor 15
GDNF	Glial-derived neurotrophic factor
GFRAL	GDNF family receptor alpha like
HIF	Hypoxia-inducible factor
HSD11B2	Hydroxysteroid 11-beta dehydrogenase 2
IHC	Immunohistochemistry
ISL1	ISL LIM homeobox 1
MAML3	Mastermind like transcriptional coactivator 3
MAPK12	Mitogen-activated protein kinase 12
MYBL2	MYB proto-oncogene like 2
NAM	Normal adrenal medulla
NES	Normalized enrichment score
NKD1	NKD inhibitor of WNT signaling pathway 1
PCA	Principal component analysis
PCC	Pheochromocytoma
PDGFR	Platelet-derived growth factor receptor
PGL	Paraganglioma
PPGL	Pheochromocytoma and paraganglioma
PTN	Pleiotrophin
RET	Ret proto-oncogene
SDH	Succinate dehydrogenase
SF-1	Steroidogenic factor 1
SFRP2	Secreted frizzled related protein 2
STAR	Steroidogenic acute regulatory protein
SYP	Synaptophysin
TBS	Tris buffered saline
TCA	Tricarboxylic acid
TCGA	The cancer genome atlas
TKI	Tyrosine kinase inhibitor
VEGFR	Vascular endothelial growth factor receptor
WNT3	Wnt family member 3
WNT4	Wnt family member 4

## Footnotes

^1^ https://github.com/UMCUGenetics/RNASeq

^2^Clinical Trials. www.ClinicalTrials.gov [accessed January 29, 2023].

^3^GEPIA 2 (Gene Expression Profiling Interacting Analysis). PTN. http://gepia2.cancer-pku.cn [Accessed January 29, 2023].
