# Supplementary material for: Whole transcriptome analysis of canine pheochromocytoma and paraganglioma
Source: Front Vet Sci. 2023 Aug 24;10:1155804. doi: 10.3389/fvets.2023.1155804 (PMC10484483; doi:10.3389/fvets.2023.1155804)
Supplement: Supplementary file 1 [file Presentation_1.PPTX]

## Slide 1
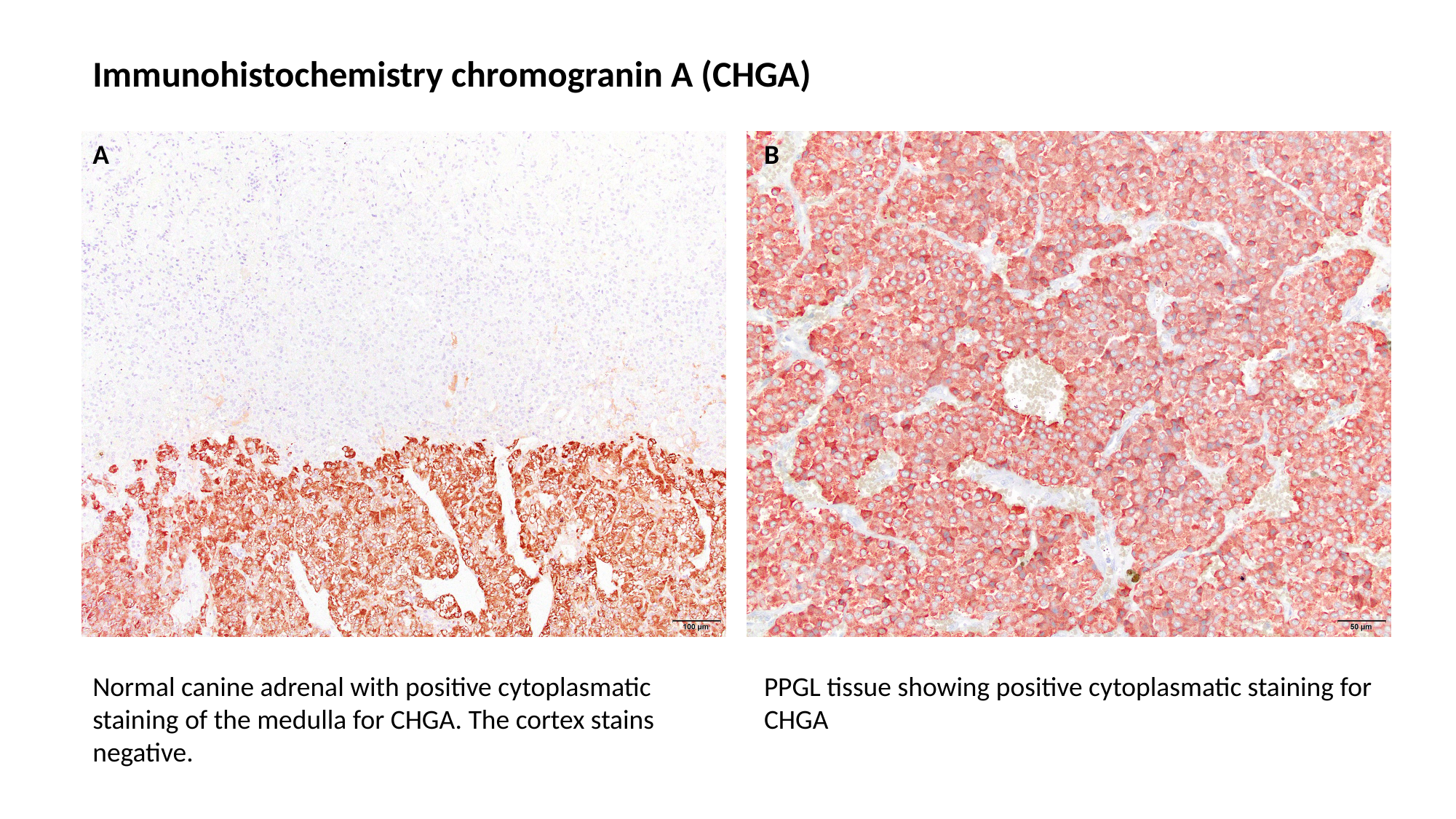

Immunohistochemistry chromogranin A (CHGA)
B
A
Normal canine adrenal with positive cytoplasmatic staining of the medulla for CHGA. The cortex stains negative.
PPGL tissue showing positive cytoplasmatic staining for CHGA

## Slide 2
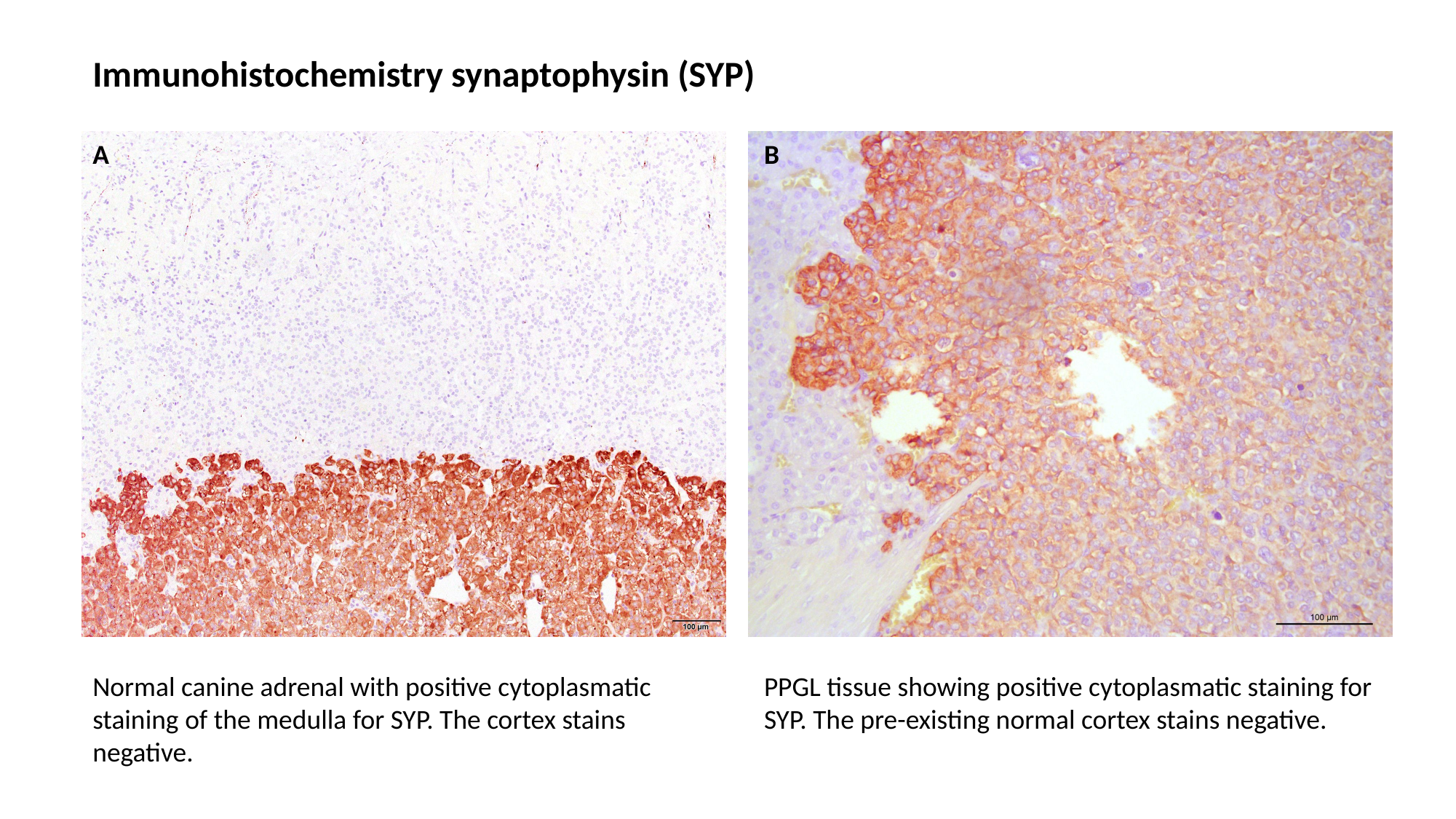

Immunohistochemistry synaptophysin (SYP)
B
A
Normal canine adrenal with positive cytoplasmatic staining of the medulla for SYP. The cortex stains negative.
PPGL tissue showing positive cytoplasmatic staining for SYP. The pre-existing normal cortex stains negative.
